# Integrated transcriptomic analysis reveals a CEBPB–DUSP1 axis driving tumor progression in colorectal cancer

**DOI:** 10.1371/journal.pone.0337892

**Published:** 2025-12-18

**Authors:** Huarong Zhou, Lili Fang

**Affiliations:** 1 Department of Medicine, Zhejiang Provincial Hospital of Dermatology, Deqing County, Zhejiang Province, China; 2 Department of Nursing, Zhejiang Provincial Hospital of Dermatology, Deqing County, Zhejiang Province, China; Tarbiat Modares University, IRAN, ISLAMIC REPUBLIC OF

## Abstract

Colorectal cancer (CRC) is a prevalent and lethal malignancy, yet the transcriptional networks driving its progression remain incompletely defined. Here, we used integrated transcriptomic analyses of healthy and colorectal cancer samples from three datasets—TCGA-COAD, GSE100179 and GSE196006, and laboratory assays to uncover CCAAT/enhancer-binding protein beta (CEBPB) as a key driver of CRC. Elevated CEBPB expression in clinical samples correlated with shorter overall survival, suggesting its utility as a prognostic marker. We next identified DUSP1, a dual specificity phosphatase critical for MAPK regulation, as a direct transcriptional target of CEBPB. Motif enrichment and promoter scans revealed three high-affinity CEBPB-binding sites in the *DUSP1* promoter. Luciferase reporter assays confirmed that CEBPB directly modulates *DUSP1* transcription. Knockdown of CEBPB in HCT116 cells rescued DUSP1 expression and reduced pro-tumor pathways linked to hyperactive MAPK. The resulting phenotype showed decreased cell proliferation, enhanced apoptosis, and partial reversion of the malignant features typically associated with CRC. These findings underscore that CEBPB–DUSP1 dysregulation contributes to CRC aggressiveness, influencing both inflammatory and metabolic processes. Overall, this work highlights CEBPB as a potential biomarker for poor prognosis and points to the CEBPB–DUSP1 axis as a promising therapeutic target. Our findings also demonstrate the power of integrated transcriptomic approaches in elucidating intricate gene regulatory networks, offering a basis for new strategies to mitigate CRC progression.

## Introduction

Colorectal cancer (CRC) is one of the most common and lethal malignancies worldwide, driven by a combination of genetic mutations, epigenetic alterations, and complex interactions within the tumor microenvironment [1,2]. Although major therapeutic advances have prolonged patient survival, the molecular mechanisms underlying CRC initiation, progression, and therapeutic resistance remain incompletely understood. Delineating these mechanisms is essential for the development of novel diagnostic markers and more effective treatment strategies. Recent studies have highlighted the crucial role of transcription factors in orchestrating gene expression programs that drive tumorigenesis [3].

The CCAAT/enhancer-binding protein beta (CEBPB) is a basic leucine zipper (bZIP) transcription factor traditionally linked to immune regulation and inflammatory responses. However, studies over the past decade have revealed multifaceted roles for CEBPB in various cancers, where it can either promote or suppress tumor growth, depending on the cellular context and interacting cofactors [4]. In breast cancer, CEBPB has been implicated in driving partial epithelial-to-mesenchymal transition (EMT), regulating tumor-promoting cytokines, and shaping the immunosuppressive microenvironment [5,6]. In lung cancer, aberrant CEBPB expression correlates with enhanced cell migration and invasion, often via crosstalk with pathways such as NF-κB [7,8]. Additionally, in hepatocellular carcinoma (HCC), CEBPB can modulate inflammation-associated oncogenesis by orchestrating cytokine production and promoting an environment conducive to tumor progression [9,10]. These studies collectively illustrate that CEBPB operates at a critical intersection between inflammation, immune signaling, and tumor cell survival, making it a compelling target for further investigation.

Within the context of colorectal cancer, accumulating evidence suggests that elevated CEBPB expression may support metastatic traits and worsen patient outcomes [11–13]. Yet, specific downstream effectors of CEBPB in CRC remain only partially elucidated. Addressing this gap is crucial, as deciphering the transcriptional network controlled by CEBPB could unlock novel biomarkers for early detection and potential therapeutic targets to mitigate disease aggressiveness.

Transcriptome‐wide integrated analysis has emerged as a powerful approach to delineate such regulatory networks. By leveraging large‐scale datasets (e.g., from The Cancer Genome Atlas) alongside in vitro and in vivo functional studies, researchers can pinpoint candidate transcription factors that drive key cancer phenotypes [14]. Unlike single‐dataset or single‐omics studies, integrated transcriptomic analyses enable cross‐validation of findings, reduce false positives, and offer a more comprehensive picture of complex gene regulatory circuits. For instance, motif enrichment within shared differentially expressed genes (DEGs) can reveal pivotal transcription factors—like CEBPB—that orchestrate multiple oncogenic pathways. Subsequent validation experiments, including luciferase reporter assays and chromatin immunoprecipitation (ChIP), help to confirm direct gene targets and quantify the functional impact of these TFs.

In the present work, we applied a multi‐layered transcriptome analysis to uncover novel target genes regulated by CEBPB in CRC. Specifically, we identified DUSP1 (dual specificity phosphatase 1) as a key downstream target and demonstrated that CEBPB binds directly to the DUSP1 promoter to modulate its transcription. DUSP1 is a major negative regulator of MAPK pathways, which are frequently dysregulated in CRC, influencing processes such as cell proliferation, stress responses, and metastasis. By exploring CEBPB‐driven transcriptional changes and correlating them with patient data, we show that this CEBPB–DUSP1 axis may contribute to CRC progression. Our study sheds light on a previously underappreciated mechanism by which CEBPB promotes tumorigenic processes, highlighting potential therapeutic avenues for intercepting this oncogenic pathway.

Overall, our findings provide fresh insight into how CEBPB shapes the tumor landscape in colorectal cancer and underscore the power of integrated transcriptomic analyses for unmasking pivotal transcription factors and their targets. By elucidating the role of the CEBPB–DUSP1 regulatory axis, we pave the way for novel therapeutic interventions aimed at rebalancing dysregulated MAPK signaling and mitigating CRC progression.

## Methods and materials

### Data sources

We analyzed healthy and colorectal cancer samples from three publicly available transcriptomic datasets: TCGA-COAD (43 healthy and 43 tumor samples), GSE100179 (20 healthy and 20 tumor samples) and GSE196006 (21 healthy and 21 tumor samples)[15,16].

RNA-seq data for colorectal cancer were obtained from The Cancer Genome Atlas (TCGA-COAD) via the Genomic Data Commons (GDC) data portal (https://portal.gdc.cancer.gov/; project ID: TCGA-COAD), and from the NCBI Gene Expression Omnibus (GEO) under accession numbers GSE196006 and GSE100179 (https://www.ncbi.nlm.nih.gov/geo/). The gene-level expression matrices used for these analyses are provided as tab-delimited supporting information (S1 Data).

### Preprocessing of raw bulk RNA-sequencing data

To analyze bulk RNA-seq data, we began with preprocessing raw sequencing reads. Quality assessment of the raw data was conducted using FastQC, which evaluates read quality and removes sequences below a defined quality threshold. Adapter trimming and removal of low-complexity sequences were performed using Trimmomatic, ensuring that only high-quality reads were retained for downstream analysis. The processed reads were aligned to the human reference genome (hg38) using the HISAT2 aligner, chosen for its efficiency in processing large datasets and its accuracy in mapping reads to the genome. After alignment, read counts per gene were obtained using HTSeq-count, which assigns reads to annotated genes based on alignment data. To account for variability in sequencing depth and gene length, we applied DESeq2 for normalization. This normalization step corrects for technical biases and ensures that gene expression comparisons across different samples reflect genuine biological variation. This workflow allowed for robust data processing and set the stage for downstream differential expression analysis.

### Differential expression analysis

To identify genes with significant expression differences between tumor and healthy samples or between experimental groups, we performed differential expression analysis on the normalized count data. The analysis was conducted using DESeq2, a statistical tool designed for RNA-seq data. *p-*values were adjusted for multiple testing using the Benjamini–Hochberg false discovery rate (FDR) procedure, and differentially expressed genes (DEGs) were defined as those with |log2FC| > 0.5 and *p*-adj < 0.05. This effect-size filter complements FDR control to prioritize biologically meaningful changes and to reduce small-effect calls that may achieve statistical significance. Consistent with these specifications, the integrated analysis across datasets identified 388 upregulated and 395 downregulated genes (S3 Table). Applying the same DESeq2 workflow and thresholds to the perturbation RNA-seq (HCT116; siCEBPB vs siNC) identified 138 upregulated and 153 downregulated DEGs (S4 Table).

### Gene Ontology (GO) analysis

Functional enrichment analysis of the identified DEGs was performed using the Metascape platform (http://metascape.org). This web-based tool was utilized to analyze Gene Ontology (GO) terms and biological pathways associated with the upregulated and downregulated genes, providing insights into the functional implications of the observed transcriptional changes in colorectal cancer.

### Tissue and cell culture

Colorectal cancer (CRC) tumor specimens (n = 30) and adjacent non-tumorous tissues (n = 28) were obtained from patients prior to receiving any therapy at the Department of Internal Medicine, Zhejiang Provincial Hospital of Dermatology, (March 6, 2024 to present). Following surgical resection, samples were immediately preserved in liquid nitrogen for downstream applications. This study involving human participants was conducted in accordance with the Declaration of Helsinki and was approved by the Institutional Ethics Committee of Zhejiang Provincial Hospital of Dermatology (Approval No. 2024-80K). All colorectal cancer tissue samples and adjacent non-tumorous tissues were collected from patients prior to any treatment.Written informed consent was obtained from all participants prior to sample collection. All collected samples were anonymized before analysis to protect patient confidentiality.

The Human CRC cell lines HCT116 were obtained from the Cell Bank (Chinese Academy of Science). Cells were cultured in standard humidified conditions at 37°C with 5% CO₂. For gene silencing experiments, small interfering RNA targeting CEBPB (siCEBPB) and a negative control (siNC) was synthesized by Qingke biotech company (Shanghai, China) and transfected into cells at a final concentration of 5 nM using Lipofectamine® 2000 (Invitrogen; Thermo Fisher Scientific, Inc., Waltham, MA, USA). The *DUSP1* promoter fragments (promoter 1: −2000 to −1500 bp, promoter 2: −1500 to −1000 bp, promoter 3: −1000–0 bp) were cloned from HCT116 and constructed into the pGL3 report vector. We performed site-directed mutagenesis using the Q5® Site-Directed Mutagenesis Kit (E0554S, New England Biolabs, Massachusetts, USA). Luciferase activity was detected using the dual-luciferase reporter assay kit (Vazyme DL101−01).

### Immunohistochemistry and quantitative real-time PCR

The tissue sectioning and staining were performed by Servicebio (Wuhan, China). Tissues were incubated overnight with an anti-CEBPB antibody (Santa Cruz, sc-56637). The IHC score was calculated as the percentage of positively stained cells.

Total tissues and cells RNA were isolated with TRIzol™ Reagent (Invitrogen). Reverse transcription was carried out with the High-Capacity cDNA Reverse Transcription Kit (Thermo Fisher, Cat. 4368814), using 5 μg of RNA in a 20 μL reaction volume. Quantitative real-time PCR (qPCR) reaction system and gene expression levels were calculated using the 2^^(-ΔΔCt)^ method as previous reported [12].

## Results

### Integrated analysis of colorectal cancer and healthy samples reveals key molecular differences

**Fig 1:**
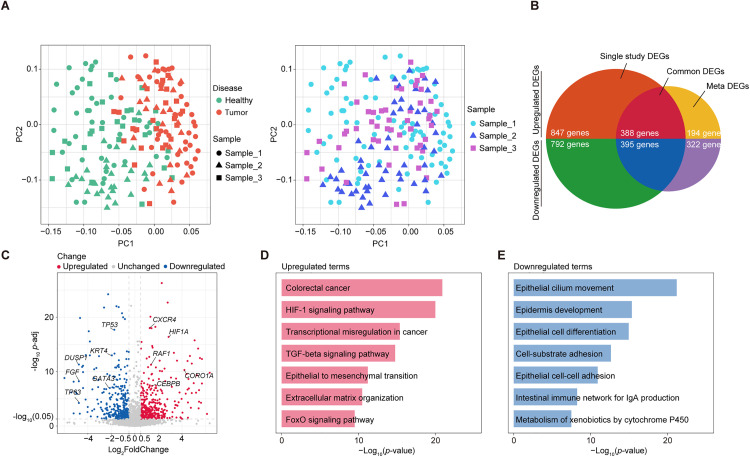
Principal Component Analysis and Differential Gene Expression in Colorectal Cancer and Healthy Samples. **(A)** Principal component analysis (PCA) of the integrated transcriptome profiles. Left: points are colored by disease status (Healthy, Tumor), and point shapes indicate sample sets (Sample_1 (TCGA-COAD), Sample_2 (GSE100179), Sample_3 (GSE196006). Right (additional panel): points are colored by sample set, and point shapes indicate disease status (Healthy, Tumor). Each point represents one sample. **(B)** Venn diagram showing the overlap between differentially expressed genes (DEGs) identified from individual studies (“Single study DEGs”) and those from the integrated meta-analysis (“Meta DEGs”). The intersection (“Common DEGs”) represents genes consistently dysregulated across all datasets. Upregulated DEGs (top half) and downregulated DEGs (bottom half) are separated by quadrants. **(C)** Volcano plot of DEGs with key overlapped genes associated with colorectal cancer biology. Each point represents a gene: red dots denote significantly upregulated genes, blue dots denote significantly downregulated genes, and grey dots indicate unchanged genes. **(D)** Bar plot of significantly enriched biological processes and pathways among upregulated DEGs enriched upregulated terms in colorectal cancer. The x-axis shows –log₁₀(*p*-adj), reflecting the enrichment significance. **(E)** Bar plot of significantly enriched biological processes and pathways among downregulated DEGs in colorectal cancer. The x-axis shows –log₁₀(*p*-adj), reflecting the enrichment significance.

Using three publicly available datasets from TCGA-COAD and GEO database (GSE196006, GSE100179) that included colorectal cancer and healthy samples, we performed an integrated analysis to uncover key molecular signatures. PCA analysis revealed that tumor and healthy groups segregated into distinct clusters, with inter-group variability exceeding intra-sample variability (Fig 1A). This finding highlights the robust molecular differences between tumor and normal tissues. Differential gene expression analysis identified 388 commonly upregulated genes and 395 commonly downregulated genes across the datasets after integrating all samples and comparing individual groups (Fig 1B, S3 Table).

Among the significantly dysregulated genes, several were particularly notable for their roles in colorectal cancer biology (Fig 1C). *CEBPB* was upregulated and has been implicated in promoting colorectal cancer progression by enhancing inflammatory responses and tumor growth signaling. *CXCR4*, a key chemokine receptor, is known to drive metastasis through increased cellular migration. The overexpression of HIF1A highlights the role of hypoxia adaptation in colorectal cancer. Additionally, *TP53* mutations, commonly associated with impaired tumor suppression, were prominently upregulated in our analysis, while the downregulation of tumor suppressor genes such as *KRT4* and differentiation-associated genes like *GATA3* points to disrupted cellular differentiation and adhesion mechanisms.

Functional enrichment analysis revealed significant upregulation of pathways and terms critical for colorectal cancer progression (Fig 1D). The colorectal cancer pathway itself was highly enriched, emphasizing the activation of canonical cancer-related signaling. HIF-1 signaling pathway suggests a central role for hypoxia adaptation and metabolic reprogramming in the tumor microenvironment. Upregulation of the TGF-beta signaling pathway and epithelial-to-mesenchymal transition (EMT) reflects enhanced tumor invasion and metastasis potential. Moreover, the enrichment of terms such as extracellular matrix organization and FoxO signaling pathway aligns with tumor microenvironment remodeling and resistance to apoptosis. FoxO family (FOXO1/3/4/6) comprises stress-responsive transcription factors that enforce cell-cycle arrest and apoptosis and are frequently curtailed by hyperactive PI3K–AKT and MAPK signaling in colorectal cancer.

Conversely, terms related to normal epithelial function and immune regulation were significantly downregulated (Fig 1E). Epithelial cilium movement, critical for maintaining epithelial barrier function, was suppressed, indicating disrupted cellular homeostasis in tumor tissues. Similarly, pathways involved in epidermis development and epithelial cell differentiation were diminished, supporting the dedifferentiated phenotype characteristic of colorectal cancer cells. Cell-substrate adhesion and epithelial cell-cell adhesion were also downregulated, consistent with EMT and reduced cellular polarity. Furthermore, immune-related pathways, such as the intestinal immune network for IgA production, showed reduced activity, suggesting immune evasion mechanisms. Finally, the metabolism of xenobiotics by cytochrome P450 was significantly decreased, potentially reflecting altered metabolic states in the tumor microenvironment.

Together, these analyses reveal key molecular alterations in colorectal cancer, highlighting critical pathways involved in tumor progression and disrupted epithelial functions. These findings provide a clear understanding of the underlying biological differences between tumor and healthy tissues, offering valuable insights for future studies.

### Transcription factor network analysis in colorectal cancer

**Fig 2:**
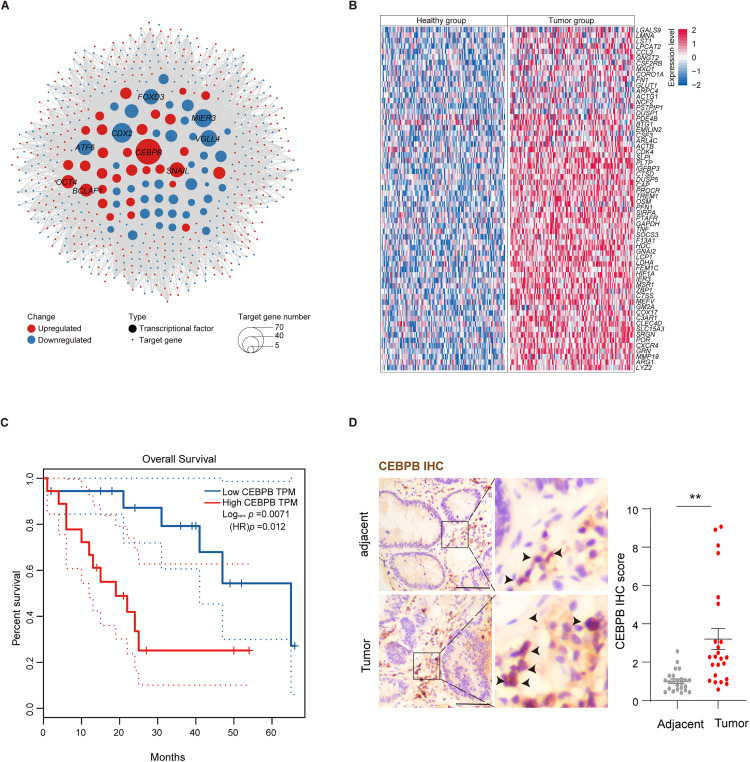
Transcription factor network and target gene expression in colorectal cancer. **(A)** Transcription factor (TF) network of the overlapped differentially expressed genes (DEGs) in colorectal cancer. Large nodes represent transcription factors, and small nodes represent their target genes. The size of each transcription factor node corresponds to the number of target genes it regulates. Red nodes indicate upregulated transcription factors or target genes, while blue nodes indicate downregulated ones. **(B)** Heatmap showing normalized expression levels of CEBPB-regulated target genes in the healthy group (left) and tumor group (right) derived from integrated transcriptomic analysis. Each column represents an individual sample, and each row corresponds to one gene. The color scale indicates relative expression values (red for high expression, blue for low expression). **(C)** Kaplan–Meier overall survival curves for colorectal cancer patients stratified by *CEBPB* transcript levels (high vs. low). Patients with high *CEBPB* expression (red line) exhibit significantly reduced survival compared to those with low expression (blue line). Log-rank test yielded *p* = 0.0071, with HR *p* = 0.012. **(D)** CEBPB IHC staining in adjacent normal and tumor colorectal tissues. The left panels show the overall field, and the right panels display magnified regions. Arrows indicate areas with positive CEBPB staining (brown signal). Scar bar = 50 μm. Tumor tissues show an increased CEBPB positive cells level compared to normal controls (n = 24). Bars represent the mean ± SEM.

We performed a transcription factor network analysis of differentially expressed genes (DEGs) to explore the molecular mechanisms driving colorectal cancer progression (Fig 2A). Among the transcription factors, *CEBPB* (CCAAT/enhancer-binding protein beta) was found to regulate the largest number of DEGs. CEBPB plays a central role in regulating immune responses, inflammation, and cellular differentiation. In colorectal cancer, upregulation of *CEBPB* is associated with increased inflammatory signaling, which contributes to tumor growth and metastasis. It also interacts with other transcription factors like NF-κB, promoting cell survival and resistance to apoptosis, which are critical for the survival and proliferation of cancer cells. Given its broad regulatory role, the upregulation of *CEBPB* in colorectal cancer suggests its significant involvement in tumorigenesis and progression.

In addition to *CEBPB*, we found upregulation of other key transcription factors such as *SNAIL*, *OCT4*, and *BCLAF1*. *SNAIL* is a well-known inducer of epithelial-to-mesenchymal transition (EMT), a process that promotes tumor invasion and migration. Its upregulation in colorectal cancer supports the metastatic potential of the disease. Similarly, *OCT4*, which is typically associated with stem cell pluripotency, plays a role in maintaining cancer stem cells in colorectal tumors, contributing to therapy resistance and relapse. *BCLAF1*, involved in regulating apoptosis, is upregulated in colorectal cancer, potentially allowing cancer cells to evade cell death, facilitating tumor progression.

Conversely, several transcription factors critical for normal epithelial function, such as *FOXD3*, *MIER3*, *VGLL4*, *CDX2*, and *ATF6*, were found to be downregulated. *FOXD3* is important for maintaining epithelial cell identity, and its downregulation may contribute to the loss of epithelial characteristics, promoting a more invasive tumor phenotype. *MIER3*, which regulates cell proliferation and apoptosis, when downregulated, could support cancer cell survival by bypassing normal growth controls. *VGLL4*, a negative regulator of the Hippo signaling pathway, is typically involved in controlling cell growth, and its downregulation could promote uncontrolled proliferation. *CDX2*, essential for intestinal epithelial differentiation, is downregulated in colorectal cancer, impairing normal differentiation processes and aiding tumor progression. Lastly, *ATF6* ad a key regulator in the term of unfolded protein response, when downregulated, may impair cellular stress responses in the tumor microenvironment, contributing to tumor cell survival.

We also examined the expression of *CEBPB*-regulated target genes (Fig 2B) and found that several of them are strongly associated with colorectal cancer biology. Among the upregulated target genes, we highlighted six that are closely linked to colorectal cancer: *LGALS9*, *GLUT1*, *IGFBP3*, *CDK4*, *DUSP1*, and *HIF1A*. *LGALS9*, a member of the galectin family, is involved in cell adhesion and migration, processes that are crucial for cancer metastasis. *GLUT1*, a glucose transporter, is upregulated to support the increased metabolic demands of cancer cells. *IGFBP3*, an inhibitor of insulin-like growth factors, regulates cell survival and apoptosis, and its upregulation may help colorectal cancer cells resist cell death. *CDK4*, a key regulator of the cell cycle, promotes cell proliferation, a hallmark of cancer. *DUSP1*, a phosphatase that regulates MAPK signaling, is involved in cellular responses to stress and inflammation, and its downregulation may repress tumor cell survival under adverse conditions. Finally, *HIF1A*, a central factor in the cellular response to hypoxia, plays a critical role in tumor growth by promoting angiogenesis and metabolic reprogramming in the hypoxic tumor microenvironment.

Interestingly, we investigated the prognostic significance of *CEBPB* in CRC and evaluated its expression in clinical specimens. Kaplan–Meier survival analysis showed that patients with high *CEBPB* transcript levels had significantly shorter overall survival compared to those with low levels (Log-rank p = 0.0071). The hazard ratio (HR = 0.012) further highlights the inverse correlation between elevated *CEBPB* expression and patient prognosis (Fig 2C). Consistent with these findings, CEBPB IHC analysis of primary tissue samples revealed markedly increased CEBPB positive cells in CRC tumors relative to normal adjacent tissues (Fig 2D). Overall, these data suggest that *CEBPB* may serve as both a potential biomarker of poor prognosis and a candidate target for novel therapeutic interventions in CRC.

### CEBPB knockdown reveals potential therapeutic targets in colorectal cancer

**Fig 3:**
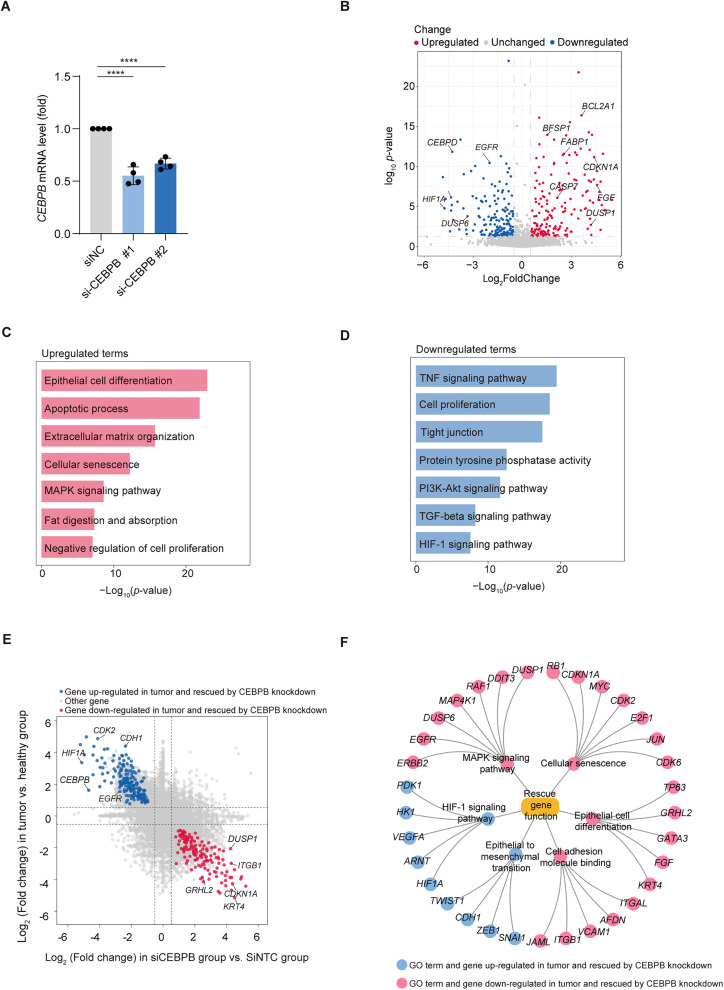
CEBPB knockdown reveals potential rescue genes and functional pathways in colorectal cancer. **(A)** Relative mRNA expression levels of *CEBPB* after CEBPB knockdown. **(B)** Volcano plot showing differentially expressed genes (DEGs) in HCT116 colorectal cancer cells after CEBPB knockdown compared with negative control (siNC). The x-axis represents the log₂(fold change) in gene expression, and the y-axis shows the –log₁₀(adjusted p-value). Red dots denote significantly upregulated genes, blue dots indicate significantly downregulated genes, and grey dots represent genes without significant change. **(C)** Bar plot of upregulated significantly enriched biological processes and pathways following CEBPB knockdown. **(D)** Bar plot of downregulated significantly enriched biological processes and pathways following CEBPB knockdown. **(E)** Scatter plot comparing log₂(fold change) in gene expression between tumor versus healthy tissues (y-axis) and between CEBPB knockdown (siCEBPB) versus control (siNC) conditions in HCT116 cells (x-axis). Each point represents one gene. Blue dots indicate genes upregulated in tumors and reversed (downregulated) upon CEBPB knockdown, while red dots represent genes downregulated in tumors and restored (upregulated) after CEBPB silencing. Grey dots denote genes without significant rescue behavior. **(F)** Functional network of rescue genes and enriched pathways. Blue nodes indicate genes upregulated in tumors and reduced by CEBPB knockdown; red nodes indicate genes downregulated in tumors and increased by CEBPB knockdown. Edges connect genes to enriched pathways; term nodes inherit the color of the predominant gene direction.

Continuing from our exploration of transcription factor networks in CRC, we conducted targeted experiments to knockdown *CEBPB* in HCT116, aiming to elucidate its pivotal role in gene regulation and pathway modulation within the tumor environment (Fig 3A). We conducted bulk RNA-seq analysis on colorectal cancer cell lines following *CEBPB* knockdown to explore its impact on gene expression (Fig 3B, S2 Data, S4 Table). The volcano plot revealed numerous significantly altered genes. Among these, genes such as *HIF1A*and *EGFR* were prominently downregulated, while *BCL2A1*, *FABP1*, *DUSP1* and *CDKN1A* were upregulated. *HIF1A*, a key factor in hypoxia adaptation, showed reduced expression, further indicating impaired tumor growth under hypoxic conditions. *EGFR*, a well-known driver of cell proliferation, was downregulated, aligning with the reduced proliferative capacity of cancer cells after CEBPB knockdown. In contrast, *CDKN1A* upregulation reinforced the cell cycle arrest observed in tumor cells, while *FABP1* and *BCL2A1* suggest metabolic and apoptotic adjustments in the post-knockdown state.

Functional enrichment analysis revealed several upregulated terms associated with tumor suppression (Fig 3C). Terms such as epithelial cell differentiation and extracellular matrix organization point to a partial restoration of epithelial homeostasis and reduced invasion potential in colorectal cancer cells. Apoptotic process was enriched, emphasizing enhanced tumor cell death following CEBPB knockdown. Cellular senescence also emerged as a significant term, reflecting the irreversible growth arrest induced in these cells. In addition, terms like tight junction and negative regulation of cell proliferation suggest a reinforcement of normal epithelial cell architecture and suppression of cancer cell growth. These findings collectively support the notion that CEBPB knockdown shifts cancer cells toward a less aggressive phenotype. In contrast, downregulated terms provided insight into the disrupted tumor-promoting pathways (Fig 3D). PI3K-Akt signaling pathway and TNF signaling pathway, both crucial for inflammation and cancer cell survival, were downregulated, further impairing tumor progression. TGF-beta signaling pathway, implicated in EMT, was suppressed, suggesting reduced metastatic potential. HIF-1 signaling pathway showed decreased activity, corroborating the reduction in hypoxia-driven adaptations.

We identified a subset of rescue genes that were dysregulated during colorectal cancer progression and reversed upon CEBPB knockdown, referred to as “rescue genes” (Fig 3E). Among these, *DUSP1*, *HIF1A*, *EGFR*, *CDKN1A*, and *KRT4* were notable. *DUSP1*, as discussed earlier, plays a pivotal role in regulating MAPK signaling. In addition to the earlier noted suppression of tumor proliferation, its re-expression upon CEBPB knockdown may also contribute to reduced inflammatory responses mediated by MAPK. *HIF1A*, while previously highlighted for its role in hypoxia, also influences metabolic reprogramming. Its downregulation here may indicate impaired glycolysis, a hallmark of tumor metabolism. *EGFR*, already recognized for its proliferative role, may also contribute to reduced cancer cell motility upon suppression. *CDKN1A*, beyond its established role in cell cycle arrest, may act synergistically with other senescence-related genes, amplifying growth inhibition. *KRT4*, previously linked to epithelial differentiation, may further promote adhesion and suppress EMT upon rescue. Functional analysis of these rescue genes revealed enrichment in processes like MAPK signaling pathway, epithelial cell differentiation, cellular senescence, and cell adhesion molecule binding (FIg 3F).

### CEBPB directly binds to the DUSP1 promoter and modulates its transcription in CRC

**Fig 4:**
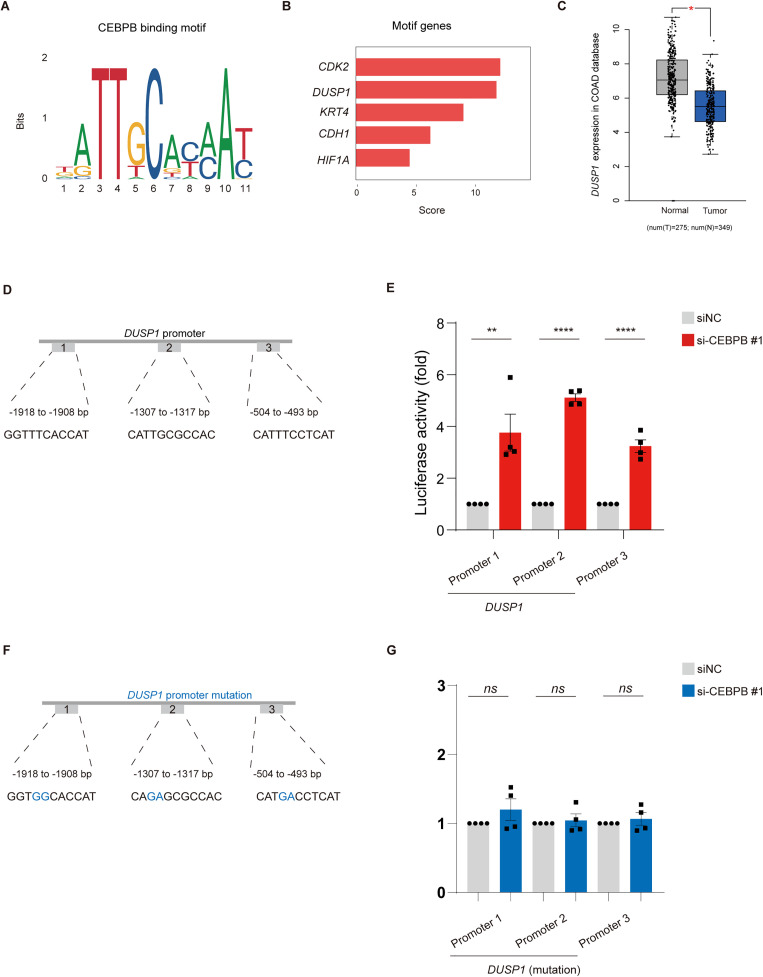
CEBPB directly binds to the DUSP1 promoter and modulates its transcription in CRC. **(A)** Sequence logo depicting the consensus CEBPB-binding motif derived from motif analysis. **(B)** Bar chart ranking top-scoring motif-associated genes (*CDK2*, *DUSP1*, *KRT4*, *CDH1*, *HIF1A*), highlighting *DUSP1* as a candidate CEBPB target. **(C)** Box plot showing DUSP1 expression in normal versus tumor tissues in the TCGA COAD database. Tumor samples display significantly lower *DUSP1* levels (*p* < 0.05). **(D)** Schematic illustration of three putative CEBPB-binding sites (Promoter 1, 2, and 3) within the *DUSP1* promoter region, spanning –2000 bp to 0 bp upstream of the transcription start site (TSS). **(E)** Luciferase reporter assays demonstrating that each *DUSP1* promoter fragment exhibits significantly increased activity (p < 0.01 or p < 0.0001) in the CEBPB knockdown compared with control. Bars represent mean ± SEM. **(F)** Site-directed mutagenesis of the three predicted *DUSP1* promoter motifs. The sequences indicate the locations and specific base substitutions of the mutated CEBPB-binding cores (highlighted in blue). **(G)** Luciferase reporter assay results of the mutated promoter constructs. Mutation of the core binding motifs abolished the transcriptional activation observed with wild-type promoters, confirming that these motifs are essential for CEBPB binding and activity. Bars represent mean ± SEM (ns, not significant).

To investigate whether CEBPB directly regulates gene expression network through transcription level, we first performed motif analysis and identified a canonical *CEBPB*-binding site enriched in several genes of interest. Notably, *DUSP1* emerged as one of the top-scoring targets [17] (Fig 4A–4B). Next, we examined *DUSP1* expression in the TCGA COAD cohort and found it to be significantly lower in tumor samples compared with normal tissues (Fig 4C). These data suggest that reduced DUSP1 levels may be linked to colorectal tumorigenesis.

To determine if CEBPB binds to and regulates the *DUSP1* promoter, we scanned the 2 kilobase upstream region of the *DUSP1* transcription start site and identified three potential CEBPB-binding motifs (Promoters 1, 2, and 3; Fig 4D). We then cloned these fragments into a luciferase reporter construct. Transient transfection assays in HCT116 cells revealed that each DUSP1 promoter fragment exhibited significant upregulation of luciferase activity in the downregulation of *CEBPB* (Fig 4E). Promoter 2 showed the strongest induction, followed by Promoters 1 and 3, consistent with CEBPB functioning as a potent transcriptional regulator of *DUSP1*. To validate whether the predicted motifs are required for CEBPB-mediated transcriptional activation, we introduced point mutations into the three core CEBPB-binding motifs within the DUSP1 promoter (Fig 4F). Luciferase assays revealed that mutation of these binding cores markedly reduced the transcriptional activation observed in wild-type promoter constructs (Fig 4G). Specifically, none of the mutant promoters exhibited significant luciferase activity changes following CEBPB knockdown, indicating that the loss of the canonical binding sites abolished the CEBPB-dependent promoter response.

These results confirm that the identified motifs are functional binding elements essential for *DUSP1* promoter activation by CEBPB. Together, these findings indicate that CEBPB directly targets the *DUSP1* promoter and modulates its transcriptional activity, thereby providing mechanistic insight into how CEBPB influences key signaling pathways in colorectal cancer.

## Discussion

In this study, we provide new evidence that CEBPB directly regulates DUSP1 at the transcriptional level and that this interaction may promote colorectal tumorigenesis. Our findings build upon prior observations that CEBPB, typically involved in inflammation and stress responses, is upregulated in CRC and correlates with poor patient survival. By identifying three conserved CEBPB-binding motifs within the *DUSP1* promoter and demonstrating significant transcriptional activation in reporter assays, we have uncovered a mechanistic basis for how CEBPB may control DUSP1 expression [18]. Our point-mutation analysis showed that altering the conserved core sequences within the predicted binding regions completely abolished promoter activation, supporting the specificity and necessity of these motifs for CEBPB-driven transcription.

Interestingly, while CEBPB often functions as a transcriptional activator, our clinical data indicate that DUSP1 expression is frequently reduced in CRC tissue samples compared to normal controls. One plausible explanation is that CEBPB’s impact on the DUSP1 promoter may depend on additional cofactors or epigenetic changes present in the tumor microenvironment. Alternatively, CEBPB could modulate DUSP1 transcription in a context-dependent manner, activating expression in some conditions while simultaneously collaborating with other oncogenic signals to repress or override DUSP1’s tumor-suppressive functions [19]. Future studies dissecting these dynamics—potentially through chromatin immunoprecipitation (ChIP) assays, histone modification profiling, or long-range chromatin interaction analyses—could help clarify the complex regulation of DUSP1 [20]. Within CRC, accumulating evidence suggests that elevated CEBPB expression supports metastatic traits and worsens outcomes. Several previous studies in colorectal cancer and other cancers have experimentally manipulated CEBPB expression, and in some cases assessed tumor growth in animal models, consistently reporting that CEBPB exerts pro-tumor effects. For instance, UBQLN4 has been shown to be activated by C/EBPB, promoting oncogenic Wnt/β-catenin signaling in CRC. In ulcerative colitis–associated colorectal cancer models, CEBPB overexpression has been tied to increased tumor growth via NF-κB/STAT3 pathway activation [21–23].

The functional significance of the CEBPB–DUSP1 axis is underscored by DUSP1’s established role in fine-tuning MAPK signaling pathways, which are critical for CRC cell survival and proliferation [24, 25]. Reduced DUSP1 levels in tumor cells may lead to hyperactivated MAPK, fueling tumor growth and metastasis. Moreover, MAPK misregulation can support an immunosuppressive tumor microenvironment and contribute to treatment resistance. By extension, re-establishing DUSP1 levels—either through CEBPB inhibition or direct DUSP1 upregulation—might represent a viable strategy to restore controlled MAPK signaling and reduce tumor aggressiveness.

Another important facet of our results lies in clinical relevance. The correlation of high CEBPB expression with unfavorable outcomes, along with the observed DUSP1 downregulation in CRC, highlights the potential of this regulatory axis as both a diagnostic and therapeutic target [26]. From a biomarker standpoint, combining CEBPB and DUSP1 expression levels may enhance prognostic accuracy. Therapeutically, small-molecule inhibitors or nucleic acid-based approaches that block CEBPB binding to the DUSP1 promoter (or that stabilize DUSP1 protein) could be explored. Additionally, since CEBPB is implicated in various inflammatory pathways, targeting the CEBPB–DUSP1 axis might also mitigate the chronic inflammation that often accompanies CRC progression [27].

Nevertheless, certain limitations of our study warrant acknowledgment. First, while our luciferase assays and motif analyses strongly suggest direct binding, confirmatory approaches such as ChIP–qPCR or genome editing (e.g., CRISPR/Cas9) would more definitively establish the functional consequences of CEBPB occupancy at these promoter sites. Second, due to the heterogeneity of CRC subtypes and microenvironments, future work should validate our findings across diverse cell lines and patient-derived xenograft models. Third, the interplay between DUSP1 and other phosphatases or oncogenic pathways could modulate the net impact on tumor progression, requiring a more comprehensive systems-level analysis to fully appreciate the therapeutic implications [28].

In summary, our work identifies a novel transcriptional relationship in which CEBPB directly regulates DUSP1 and proposes that dysregulation of this axis contributes to the aggressive phenotype of CRC. These insights not only clarify the functional complexity of CEBPB in colorectal cancer but also pave the way for new therapeutic strategies aimed at restoring controlled MAPK signaling. Future efforts will focus on verifying these findings in larger patient cohorts, exploring combination therapies that target multiple oncogenic nodes, and determining whether modulating CEBPB–DUSP1 activity can meaningfully improve clinical outcomes for patients with advanced CRC.

## Supporting information

S1 Data Public cohorts expression matrices (TCGA-COAD, GSE100179, GSE196006). Tab-delimited matrix (genes × samples) with RPKM values. Gene identifiers are HGNC-approved gene symbols. Columns correspond to individual samples. This file enables full reproduction of the cross-cohort analyses.
(TXT)

S2 Data Perturbation RNA-seq expression matrix (HCT116, siCEBPB vs siNC). Tab-delimited matrix (genes × samples) with RPKM values. Gene identifiers are HGNC-approved gene symbols. Columns include group labels (siCEBPB/siNC) and replicate IDs.
(TXT)

S3 Table Differential expressed genes between tumor and healthy groups. (XLSX)

S4 Table Differential expressed genes between si-CEBPB and si-NC HCT116 cells. (XLSX)

## References

[1] Armaghany T, Wilson JD, Chu Q, Mills G. Genetic alterations in colorectal cancer. Gastrointest Cancer Res. 2012;5(1):19–27. 22574233 PMC3348713

[2] Li Q, Geng S, Luo H, Wang W, Mo Y-Q, Luo Q, et al. Signaling pathways involved in colorectal cancer: pathogenesis and targeted therapy. Signal Transduct Target Ther. 2024;9(1):266. doi: 10.1038/s41392-024-01953-7 39370455 PMC11456611

[3] Bode AM, Zhang T. Recent Advances in Carcinogenesis Transcription Factors: Biomarkers and Targeted Therapies. Cancers (Basel). 2023;15(19). 10.3390/cancers15194673 PMC10571516 37835367

[4] Hartl L, Duitman J, Bijlsma MF, Spek CA. The dual role of C/EBPδ in cancer. Crit Rev Oncol Hematol. 2023;185:103983. doi: 10.1016/j.critrevonc.2023.103983 37024021

[5] Matherne MG, Phillips ES, Embrey SJ, Burke CM, Machado HL. Emerging functions of C/EBPβ in breast cancer. Front Oncol. 2023;13:1111522. doi: 10.3389/fonc.2023.1111522 36761942 PMC9905667

[6] Qi L, Sun B, Yang B, Lu S. CEBPB regulates the migration, invasion and EMT of breast cancer cells by inhibiting THBS2 expression and O-fucosylation. Hum Mol Genet. 2023;32(11):1850–63. doi: 10.1093/hmg/ddad022 36728807

[7] Chen W, Li Z, Bai L, Lin Y. NF-kappaB in lung cancer, a carcinogenesis mediator and a prevention and therapy target. Front Biosci (Landmark Ed). 2011;16(3):1172–85. doi: 10.2741/3782 21196225 PMC3032584

[8] Okazaki K, Anzawa H, Katsuoka F, Kinoshita K, Sekine H, Motohashi H. CEBPB is required for NRF2-mediated drug resistance in NRF2-activated non-small cell lung cancer cells. J Biochem. 2022;171(5):567–78. doi: 10.1093/jb/mvac013 35137113

[9] Tseng H-H, Hwang Y-H, Yeh K-T, Chang J-G, Chen Y-L, Yu H-S. Reduced expression of C/EBP alpha protein in hepatocellular carcinoma is associated with advanced tumor stage and shortened patient survival. J Cancer Res Clin Oncol. 2009;135(2):241–7. doi: 10.1007/s00432-008-0448-5 18663475 PMC12160288

[10] Zhao X, Reebye V, Hitchen P, Fan J, Jiang H, Sætrom P, et al. Mechanisms involved in the activation of C/EBPα by small activating RNA in hepatocellular carcinoma. Oncogene. 2019;38(18):3446–57. doi: 10.1038/s41388-018-0665-6 30643190

[11] Mi L, Hu K, Wen X, Sun J, Wu A, Wang M, et al. Upregulation of C/EBPα contributes to colorectal cancer growth, metastasis and indicates poor survival outcome. Am J Cancer Res. 2018;8(8):1449–65. 30210916 PMC6129490

[12] Sun D, Wang C, Long S, Ma Y, Guo Y, Huang Z, et al. C/EBP-β-activated microRNA-223 promotes tumour growth through targeting RASA1 in human colorectal cancer. Br J Cancer. 2015;112(9):1491–500. doi: 10.1038/bjc.2015.107 25867276 PMC4453668

[13] Zhang Y, Li L, Chu F, Wu H, Xiao X, Ye J, et al. Itraconazole inhibits tumor growth via CEBPB-mediated glycolysis in colorectal cancer. Cancer Sci. 2024;115(4):1154–69. doi: 10.1111/cas.16082 38278779 PMC11007002

[14] Cancer Genome Atlas Research Network, Weinstein JN, Collisson EA, Mills GB, Shaw KRM, Ozenberger BA, et al. The Cancer Genome Atlas Pan-Cancer analysis project. Nat Genet. 2013;45(10):1113–20. doi: 10.1038/ng.2764 24071849 PMC3919969

[15] Marx OM, Mankarious MM, Eshelman MA, Ding W, Koltun WA, Yochum GS. Transcriptome Analyses Identify Deregulated MYC in Early Onset Colorectal Cancer. Biomolecules. 2022;12(9):1223. doi: 10.3390/biom12091223 36139061 PMC9496520

[16] Szigeti KA, Kalmár A, Galamb O, Valcz G, Barták BK, Nagy ZB, et al. Global DNA hypomethylation of colorectal tumours detected in tissue and liquid biopsies may be related to decreased methyl-donor content. BMC Cancer. 2022;22(1):605. doi: 10.1186/s12885-022-09659-1 35655145 PMC9164347

[17] Castro-Mondragon JA, Riudavets-Puig R, Rauluseviciute I, Lemma RB, Turchi L, Blanc-Mathieu R, et al. JASPAR 2022: the 9th release of the open-access database of transcription factor binding profiles. Nucleic Acids Res. 2022;50(D1):D165–73. doi: 10.1093/nar/gkab1113 34850907 PMC8728201

[18] Shen J, Zhou S, Shi L, Liu X, Lin H, Yu H, et al. DUSP1 inhibits cell proliferation, metastasis and invasion and angiogenesis in gallbladder cancer. Oncotarget. 2017;8(7):12133–44. doi: 10.18632/oncotarget.14815 28129656 PMC5355331

[19] Gil-Araujo B, Toledo Lobo M-V, Gutiérrez-Salmerón M, Gutiérrez-Pitalúa J, Ropero S, Angulo JC, et al. Dual specificity phosphatase 1 expression inversely correlates with NF-κB activity and expression in prostate cancer and promotes apoptosis through a p38 MAPK dependent mechanism. Mol Oncol. 2014;8(1):27–38. doi: 10.1016/j.molonc.2013.08.012 24080497 PMC5528511

[20] Gade P, Kalvakolanu DV. Chromatin immunoprecipitation assay as a tool for analyzing transcription factor activity. Methods Mol Biol. 2012;809:85–104. doi: 10.1007/978-1-61779-376-9_6 22113270 PMC3891665

[21] Huang T, Zhang Y, Wu Y, Han X, Li L, Guo Z, et al. CEBPB dampens the cuproptosis sensitivity of colorectal cancer cells by facilitating the PI3K/AKT/mTOR signaling pathway. Saudi J Gastroenterol. 2024;30(6):381–8. doi: 10.4103/sjg.sjg_169_24 39246119 PMC11630481

[22] Ma Y, Chen Y, Zhan L, Dong Q, Wang Y, Li X, et al. CEBPB-mediated upregulation of SERPINA1 promotes colorectal cancer progression by enhancing STAT3 signaling. Cell Death Discov. 2024;10(1):219. doi: 10.1038/s41420-024-01990-9 38710698 PMC11074302

[23] Tang X, Liang Y, Sun G, He Q, Qu H, Gao P. UBQLN4 is activated by C/EBPβ and exerts oncogenic effects on colorectal cancer via the Wnt/β-catenin signaling pathway. Cell Death Discov. 2021;7(1):398. doi: 10.1038/s41420-021-00795-4 34930912 PMC8688525

[24] Bahar ME, Kim HJ, Kim DR. Targeting the RAS/RAF/MAPK pathway for cancer therapy: from mechanism to clinical studies. Signal Transduct Target Ther. 2023;8(1):455. doi: 10.1038/s41392-023-01705-z 38105263 PMC10725898

[25] Grossi V, Peserico A, Tezil T, Simone C. p38α MAPK pathway: a key factor in colorectal cancer therapy and chemoresistance. World J Gastroenterol. 2014;20(29):9744–58. doi: 10.3748/wjg.v20.i29.9744 25110412 PMC4123363

[26] Tang Z, Li C, Kang B, Gao G, Li C, Zhang Z. GEPIA: a web server for cancer and normal gene expression profiling and interactive analyses. Nucleic Acids Res. 2017;45(W1):W98–102. doi: 10.1093/nar/gkx247 28407145 PMC5570223

[27] Smith MP, Sanchez-Laorden B, O’Brien K, Brunton H, Ferguson J, Young H, et al. The immune microenvironment confers resistance to MAPK pathway inhibitors through macrophage-derived TNFα. Cancer Discov. 2014;4(10):1214–29. doi: 10.1158/2159-8290.CD-13-1007 25256614 PMC4184867

[28] Shen J, Zhang Y, Yu H, Shen B, Liang Y, Jin R, et al. Role of DUSP1/MKP1 in tumorigenesis, tumor progression and therapy. Cancer Med. 2016;5(8):2061–8. doi: 10.1002/cam4.772 27227569 PMC4884638

